# Current Outcomes of Laparoscopic Duodenal Switch

**DOI:** 10.1186/s13022-016-0024-7

**Published:** 2016-01-21

**Authors:** Laurent Biertho, Frédéric Simon-Hould, Simon Marceau, Stéfane Lebel, Odette Lescelleur, Simon Biron

**Affiliations:** Department of Bariatric Surgery, Quebec Heart and Lung Institute, Laval University, Quebec, Canada; Institut Universitaire de Cardiologie et de Pneumologie de Québec, 2725, Chemin Ste-Foy, Quebec, QC G1V 4G5 Canada

**Keywords:** Bariatric surgery, Biliopancreatic diversion, Duodenal switch, Laparoscopy

## Abstract

**Background:**

Biliopancreatic diversion with duodenal switch (BPD-DS) has long been considered as the bariatric procedure with the highest peri-operative and long-term complication rate. However, modern peri-operative care, including laparoscopic and staged-approach, has significantly reduced the complication rate related to this procedure. The goal of this article is to provide an overview of the current outcomes of laparoscopic BPD-DS in a high volume centre.

**Methods:**

All patients who had a laparoscopic BPD-DS with a hand-sewn anastomosis performed between 2011 and 2015 (N = 566) were reviewed. Data were obtained from our prospectively maintained electronic database and are reported as a Mean ± standard deviation.

**Results:**

The mean age of the 566 patients was 41 ± 10 years, with 78 % women. Initial body mass index was 49 ± 6 kg/m^2^. There was no 90-days mortality. Hospital stay was 4.5 ± 3 days. Major 30-days complications occurred in 3.0 % (n = 17) of the patients and minor complications in 2.5 % (N = 14). Excess weight loss was 81 ± 14 % at 12 m, 88 ± 13 % at 24 m, 83 ± 14 % at 36 months. Total body weight loss (kg) was 57 ± 13 kg at 12 months, 63 ± 14 kg at 24 months and 61 ± 17 kg at 36 months. Hemoglobin A1C (HbA1C) dropped from 6.1 ± 1 % to 4.7 ± 0.5 % (p < 0.005) and the percentage of patients with an HbA1C above 6 % decreased from 38 to 1.4 % (p < 0.005). Over 21 ± 12 months follow-up, readmission was required in 3.5 % and reoperation in 0.5 % of the patients.

**Conclusion:**

The current short and medium-term complication rate of laparoscopic BPD-DS are similar to other mixed bariatric procedures with excellent metabolic outcomes.

## Background

Biliopancreatic Diversion (BPD) was first described by Nicola Scopinaro in 1979 [1]. This technique combined an horizontal gastric resection with closure of the duodenal stump, a gastro-ileostomy and an ileo-ileostomy, to create a 50-cm common channel and a 250-cm alimentary channel. The technique was modified to a Duodenal Switch procedure, based on the procedure described by Dr DeMeester, to treat bile gastritis in the late 80’s [2]. It evolved in the following years to the “modern” BPD-DS procedure, which includes a partial gastrectomy performed along the greater curvature (i.e., a Sleeve Gastrectomy, SG), transection of the duodenum 3–4 cm distal to the pylorus, and creation of a 250 cm alimentary limb [3, 4]. The biliary limb is anastomosed 100 cm from the ileo-caecal valve, to create the common channel. The malabsorptive component of the procedure results from the separation of food from the bile and pancreatic juice. This results in a reduction of caloric and food absorption, particularly of lipids, and metabolic changes through modifications of incretins levels. The excellent long-term metabolic outcomes of the procedure have never really be questioned. Nevertheless, Buchwald et al. [5] reported that Bilio-Pancreatic Diversion represented only 2 % of the 344,221 bariatric surgeries performed worldwide in 2008. There are multiple reasons for his finding, including the increased technical complexity, high complication and mortality rates reported in the literature and increased risk of protein malnutrition [6, 7]. However, there has been a number of significant improvements in the peri-operative management of morbidly obese patients since the first report of laparoscopic BPD-DS in 1999 [8] and exposure to this procedure has become of outmost importance for the management of failure after sleeve gastrectomy.

The goal of this study is to describe the current morbidity and medium term outcomes of BPD-DS in a tertiary care center specialized in this technique.

## Methods

All patients who had a laparoscopic BPD-DS using a hand-sewn anastomosis at the Quebec heart and lung institute, a university affiliated tertiary care center, were included in this study. open BPD-DS has been performed since 1989 in our Institution. Indication for surgery follows the standard 1991 NIH recommendations [9]. Laparoscopic BPD-DS was introduced in November 2006, and we first used a mechanical stapler for the duodenal anastomosis (21-mm circular stapled anastomosis). This technique was associated with a higher rate of complication (including stenosis, leak, bleeding) as was reported previously [10]. We transitioned to hand-sewn anastomosis, which has been our standard operative technique since 2011. All patients who were operated using this technique were included in this study, up to February 2015. Data were extracted from a prospectively maintained electronic database and reviewed retrospectively.

### Patients selection

Patient selection followed the standard NIH recommendations for bariatric surgery [10]. All patients were assessed by a bariatric surgeon, dietician, nurse specialized in bariatric surgery and social worker. Patients had an electrocardiogram, chest X-ray, blood work, sleep apnea testing and consultation in pneumology. Vitamins or minerals supplementation were started when deficiencies were discovered before the surgery. Consultation with a psychiatrist was requested when patient had a history of psychiatric disease. Standard pre-operative education specific to BPD-DS or sleeve gastrectomy was given to all patients. Nutritional deficiencies were treated before surgery according to standard supplementation protocols.

### Surgical technique

All patients followed our routine pre-operative preparation including a low-residue diet for 2 days before surgery, antibioprophylaxis (Cefazolin 2–3 g at the time of surgery) and thrombo-prophylaxis (standard or low-molecular weight subcutaneous heparin). A 15-mm Hg pneumo-peritoneum is first created. The greater curvature of the stomach is mobilized using ultrasonic shears (Ace Ultrasonic, Ethicon EndoSurgery, Cincinnati, OH, USA). A 34–44 Fr Bougie is used for the calibration of the sleeve. The stomach is then transected along that Bougie using an articulating linear stapler-cutter (Echelon-Flex long 60, Ethicon EndoSurgery, Cincinnati, OH, USA), staring 7–8 cm from the pylorus, to create a gastric reservoir, with an estimated volume of 250 cc. The duodenum is then transected 3–4 cm from the pylorus, using a blue cartridge. The ileo-caecal valve is then identified and the small bowel is transected 250 cm proximal, using a white cartridge. The duodeno-ileal anastomosis is then created. A hand-sewn anastomosis, using two posterior layers and one anterior layer of absorbable sutures (3-0 V-lock© suture). The mesenteric window is closed using a 2-0 Prolene suture. Routine cholecystectomy was also performed.

Standard post-operative orders are used, including ulcer prevention, thrombo-prophylaxis and feeding protocol. Patients were discharged when tolerating a soft diet, with vitamins and minerals daily supplementations. Patients received a multivitamin complex (Centrum Forte), vitamin A 20.000 IU, vitamin D 50.000 IU, calcium carbonate 1000 mg and ferrous sulfate 300 mg.

### Follow-up

Patients were followed at the clinics at 4, 8, 12, 18 and 24 months post-op and yearly thereafter. Blood analyses were performed at these times, including a complete blood count, electrolytes, urea and creatinine, calcium, parathormone levels, vitamin D, vitamin A, serum iron, total iron binding capacity and ferritin. Supplements were adjusted over time according to these analyses using standardized supplementation protocols. The percentage of excess weight loss (EWL) was calculated as followed: (initial weight–current weight)/(initial weight–ideal weight). The ideal weight was calculated by multiplication the square of the patient’s height in meters by 23. The body mass index (BMI) was calculated by dividing the patient’s weight in kilograms by the square of the height in meters.

### Statistical methods

The data are reported as the mean ± standard deviation for continuous data or as percentages for categorical variables. Statistical analysis was performed using a Student’s t test for continuous variables, and the Pearson’s Chi square test for categorical variables, except when a low number of observations required Fisher’s exact test. p < 0.05 was considered statistically significant.

## Results

The demographic data for the 566 patients is described in Table 1. Initial BMI was 49 ± 6.1 kg/m^2^. All patients underwent a laparoscopic BPD-DS in our Institution, by four different surgeons. One patient required conversion to open surgery because of difficulties getting good exposure and was kept in the series in an intention-to-treat process. Post-operative complications are described in Table 2. Major complications occurred in 3 % of the patients and reoperation was required in 1.9 % of the patients. A leak occurred at the duodenal anastomosis in 0.7 % of the patients (n = 4) and at the gastric level in 0.2 % (n = 1). There was no short- or medium-term mortality, during a mean 21 ± 12 months follow-up. During that period, readmission for a medical problem related to the surgery was required in 3.5 % of the population, and a reoperation was required in 0.5 % (Table 3), including two patients who required a surgical revision for malnutrition.

**Table 1:** Demographic data

	BPD-DS
N	566
Age (years)	41 ± 9.5
% female patients	78 %
BMI (kg/m^2^)	49 ± 6.1
Weight (kg)	135 ± 22
Waist diameter (cm)	129 ± 35
Hip diameter (cm)	136 ± 37
Type II diabetes % (n)	50 % (282)
Hypertension, % (n)	46 % (259)
Sleep Apnea, % (n)	60 % (342)
Dyslipidemia, % (n)	30 % (171)
Number of comorbidities	4.4 ± 1.9

Data are expressed as the mean ± standard deviation for continuous data and as percentages for categorical data

**Table 2:** Peri-operative data

	BPD-DS
N	566
Operative time	199 ± 43
Blood loss	37 ± 49
Length of stay	4.5 ± 3.2
Mortality	0
Conversion	1 (0.2 %)

Data are expressed as the mean ± standard deviation for continuous data and as percentages for categorical data

**Table 3:** Thirty-days complications

Variable	Percentage (N)	Required reoperation
Major complications (N = 566)		
Duodenal leak	0.7 (4)	0.7 (4)
Gastric leak	0.2 (1)	–
Intra-abdominal abscess	0.5 (3)	0.2 (1)
Pulmonary embolism	0.2 (1)	–
Myocardial infarction	0.2 (1)	–
Other	0.4 (2)	0.2 (1)
Obstruction	0.5 (3)	0.5 (3)
Digestive bleeding	0.4 (2)	0.4 (2)
Total	3.0 (17)	1.9 (11)
Minor complications		
Pneumonia	0.4 (2)	
Food intolerance	1 (6)	
Stenosis	0.2 (1)	
Atelectasis	0.2 (1)	
C Difficile colitis	0.2 (1)	
Pancreatitis	0.2 (1)	
Wound infection	0.4 (2)	
Total	2.5 (14)	
Grand total	5.5 (31)	1.9 (11)

Data are presented as a percentage (number of cases)

**Fig. 1:**
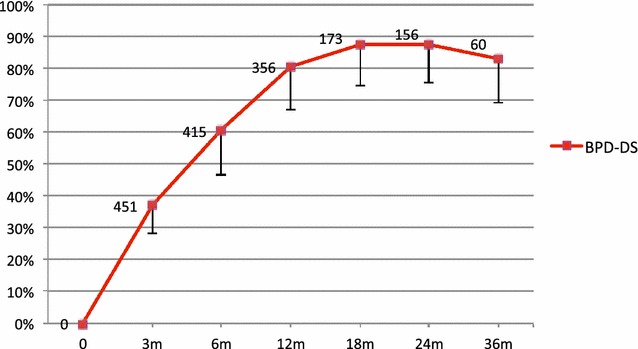
Percentage of excess weight loss over time. Data are reported as the Mean ± standard deviation. Numbers above the curve represents the number of available data at each interval

Figure 1 summarizes the percentage of EWL. EWL at three years (n = 60) was 83 ± 14 %, which corresponds to a total body weight loss of 61 ± 17 kg. At that time, one patient (1.7 %) had a BMI above 35 kg/m^2^, three patients (5 %) had a BMI between 30 and 35 kg/m^2^, and 14 (23 %) had a BMI between 25 and 30 kg/m^2^.

Tables 4 and 5 summarizes some of the biological values, comparing data before to after surgery, at the time of last available follow-up (mean 21 ± 12 months). Before surgery, 38 % of the population had an Hemoglobin A1C (HbA1C) above 6 %. After surgery, only 1.4 % had an HbA1C above 6 % (p < 0.005). There was also a significant drop in total cholesterol, low density lipoproteins and triglycerides. There was a significant improvement in ferritin level, vitamin D-25-OH and vitamin B12. However, albumin, hemoglobin and vitamin A dropped significantly. Even though the mean albumin level dropped significantly, values were below normal (35 gr/l) in only two patients at 2 years (2/182, 1.1 %) and one patient at 3 years (1/70 or 1.4 %).

**Table 4:** Medium-term complications

Variable	Percentage (N)	Required reoperation
Denutrition	1.8 (10)	0.4 (2)
Small bowel obstruction	0.5 (3)	
Food intolerance	0.5 (3)	
Gallstones	0.2 (1)	1
Other	0.5 (3)	
Total	3.5 % (20)	0.5 % (3)

Data are presented as a percentage (number of cases)

**Table 5:** Changes in some biological values over time

Variable	Before surgery	After surgery	p
Hemoglobin A1C (%, n = 473)	6.1 ± 1	4.7 ± 0.5	<0.005
Above six (%, n)	38 % (216)	1.4 % (8)	
Fasting plasma glucose (mmol/l, n = 236)	6.57 ± 2.1	4.7 ± 0.9	<0.005
Total cholesterol (mmol/l, n = 236)	4.47 ± 0.9	3.2 ± 0.6	<0.005
HDL (mmol/l, n = 235)	1.22 ± 0.29	1.22 ± 0.29	NS
LDL (mmol/l, n = 233)	2.54 ± 0.79	1.55 ± 0.49	<0.005
Triglycerides (mmol/l, n = 233)	1.59 ± 0.8	1.0 ± 0.4	<0.005
Total cholesterol/HDL	3.79 ± 1.0	2.8 ± 0.8	<0.005
Hemoglobin (g/l, n = 506)	135 ± 11	128.5 ± 13	<0.005
Ferritin (ng/ml, n = 477)	132 ± 153	157 ± 131	<0.005
Albumin (g/l, n = 502)	42.3 ± 2.45	39.9 ± 3.8	<0.005
Vitamin A (µmol/l, n = 394)	1.9 ± 0.45	1.53 ± 0.4	<0.005
Vitamin D-25 (mmol/l, n = 443)	51.6 ± 24	94.3 ± 39	<0.005
Vitamin B12 (pmol/l, n = 479)	300 ± 120	401 ± 160	<0.005

## Discussion

The excellent long-term weight loss and correction of obesity-related diseases after BPD-DS have never been really challenged. In a meta-analysis of the bariatric literature, Buchwald et al. [9] reported that BPD is the surgery offering the best long-term EWL (70.1 %) and improvement in type 2 diabetes (98 %). However, BPD has also been associated in the past, with some of the highest mortality rate (1.1 % compared with 0.28 % for all procedures). More recently, modern peri-operative care, the use of minimally invasive technique and staged approaches have allowed a reduction of the mortality rate to those observed after other bariatric surgeries [11]. Indeed, we observed a mortality rate of 0.1 % in a series of 1000 BPD-DS which included our initial cases of laparoscopic BPD-DS and a significant portion of open DS [10]. In this series, we did not experience any mortality in a consecutive series of 566 patients.

In that series of patients, which included 228 laparoscopic cases and 772 open cases [10], the major complication rate was 7.4 versus 3.0 % in the present series (p = 0.0002) and the minor complication rate was 9.1 versus 5.5 % (p = 0.01). The risk of leak using a circular-stapled anastomosis was 2.6 %, while the use of a hand-sewn technique allowed to reduce that risk 0.4 %, which is consistent with the leak rate reported in recent series of gastric bypasses [12]. In addition, the use of a hand-sewn technique has allowed to virtually eliminate the risk of anastomotic stenosis, which occurred in an average of 10 % of patients who had a circular-stapled anastomosis.

The limitations of this study include its retrospective nature. Even though data were entered prospectively into our database, some complications might have been missed. In addition, we looked at short term outcomes and we do not have the 5–10 year data on laparoscopic technique to discuss the long-term metabolic changes. However, we believe that the technical changes related to the laparoscopic approach should not impact the good long-term outcomes we reported previously, and could even be beneficial in decreasing the risk of ventral hernia, small bowel obstruction and complications related to any abdominal reoperation. In addition, we did not report the changes in quality of life or side-effects related to the procedure.

## Conclusion

In experienced hands, laparoscopic BPD-DS is only slightly more technically difficult than other bariatric procedures, like RYGB. The rate of major peri-operative complications is low, at 3 %, which is in similar ranges compared to other bariatric procedures. In addition, BPD-DS offers some of the best weight loss and cure-rate of obesity related diseases. It also allows a better eating experience, by preserving the pyloric valve and avoiding dumping syndrome. These long-term benefits come at the cost of certain gastrointestinal side effects and long-term compliance with vitamin supplementation.
